# Post-Mortem Examinations in Asylums

**Published:** 1900-05-19

**Authors:** 


### Editor's Letter-Box.

[Our Correspondents are reminded that prolixity is a great bar to publication, and that brevity of style and conciseness of statement greatly facilitate early insertion.]

### POST-MORTEM EXAMINATIONS IN ASYLUMS.

Sir,—Your issue of the 12th inst. contains a letter from Dr. Kidd, with an editorial note appended, in answer to which I trust you will allow me to quote a sentence written by Dr. Wallis, late Commissioner in Lunacy, and to add a word or two of my own. "The usual pathological work in asylums," said Dr. Wallis, "is so much confined to the examination of the utterly wasted and degenerated brains of the general paralytics, chronically disorganised and senile brains, and confirmed epileptics. What we more particularly want is the careful and exhaustive examination by many workers of any brain which may be available from the unexpected, sudden, or early death of a general paralytic in the first stage; a recent case of melancholia or acute mania dying from pneumonia, or some sudden complication or accidental occurrence." This suggestion of Dr. Wallis's could be well carried out by holding post-mortem examinations in 5 per cent., or at most 10 per cent., of the deaths. But, sir, this is not the boast of some of our latter-day medical superintendents, who plume themselves on making these examinations in every case of death. The whole question was threshed out some twenty years' since in the *Journal of Mental Science*, and I do not think any more need be said on one aspect of it, at any rate. I am glad to learn that consent of the relatives is first obtained at the Sussex Asylum. But how does Dr. Kidd obtain this? Notices of death must be sent to the authorities within 48 hours. If Dr. Kidd considers it his duty to make these examinations, he is subjectively right in so doing; but it is not improbable that the public and his own committee will one day take a different view. To adduce the detection of supposed injuries as one reason for these examinations is surely playing pathological studies rather low down.

An Old Asylum Medical Officer.

